# Combination of PSA density and MLR improves the diagnostic accuracy of prostate cancer

**DOI:** 10.3389/fonc.2025.1570584

**Published:** 2025-07-16

**Authors:** Feng Guo, Aerken Maolake, Zecheng Ni, Xun Li, Bide Liu, Zetian Tang, Zhenfeng Shi, Jiuzhi Li

**Affiliations:** ^1^ Department of Urology, People’s Hospital of Xinjiang Uygur Autonomous Region, Xinjiang, China; ^2^ Departments of Cancer Genetics, Roswell Park Comprehensive Cancer Center, Buffalo, NY, United States

**Keywords:** PSA density, MLR, diagnosis, prostate cancer, inflammatory markers, ROC, predictive value

## Abstract

Prostate-specific antigen (PSA) is used to screen for prostate cancer for decades. However, PSA has poor specificity in prostate cancer screening within the 4.0- to 10.0-ng/mL range. This study aimed to develop a new prediction model for PCa in patients with a PSA level of 2.5–20 ng/mL. The clinical data of 80 patients with PSA 4–22 ng/mL from 2016 to 2022 were selected for retrospective analysis. Prostate volume was estimated by suprapubic ultrasonography. PSA and the inflammatory markers like neutrophil-to-lymphocyte ratio (NLR), platelet-to-lymphocyte ratio (PLR), and monocyte-to-lymphocyte ratio (MLR) in peripheral blood were analyyzed to assess their value in PCa. The diagnostic performance of PSA, PSA density (PSAD), and inflammatory markers, respectively, was estimated by ROC curve. The areas under the ROC curve for f/t PSA, PV, PSAD, MLR, NLR, and PLR for predicting PCa in patients with a PSA level of 4.0–22.0 ng/mL were 0.7375, 0.7774, 0.8294, 0.5945, 0.5571, and 0.5437, respectively. The PSAD performed better than f/t PSA and PV in the diagnosis of PCa. The specificity of PSAD was higher than that of f/tPSA when tPSA was in the gray zone (between 4 and 10 ng/mL). The area under the curve (AUC) increased when PSAD was combined with MLR in patients with PSA 4–10 ng/mL and patients with PSA 10–22 ng/mL, and the positive predictive values were 81.81% and 90.91%, respectively (*P* = 0.0008 and *P* = 0.0002). PSAD has a moderate diagnostic value for PCa detection. The combination of PSAD and MLR could improve the diagnostic accuracy in PCa diagnosis.

## Introduction

Prostate cancer (PCa) remains one of the most frequently diagnosed malignancies in men globally and contributes significantly to cancer-related morbidity and mortality. In the United States alone, projections for 2023 indicated approximately 288,300 new diagnoses and 34,700 deaths attributed to PCa (1, 2).

Since the late 1980s, the widespread implementation of prostate-specific antigen (PSA) testing has facilitated the earlier detection of PCa (3). PSA remains a cornerstone biomarker; however, its limited specificity presents diagnostic challenges. Non-malignant conditions such as benign prostatic hyperplasia (BPH) and prostatitis can also elevate the PSA levels, particularly within the so-called “gray zone” (2.0–10.0 ng/mL), where the PSA levels for benign and malignant conditions often overlap (4–6). To improve specificity and reduce unnecessary biopsies, additional PSA-derived indices such as the percentage of free PSA (f/tPSA), PSA density (PSAD), and free PSA density (fPSAD) have been proposed (7–9). Despite these efforts, inconsistencies remain—for instance, while f/tPSA has shown value in Western populations, recent findings from China suggest that it may not enhance PCa detection in patients with PSA levels ranging from 2.5 to 20.0 ng/mL (10). Notably, the detection rates of PCa within PSA ranges of 2.0–4.0 ng/mL remain nontrivial, reported at approximately 26% in American and 23.6% in Japanese cohorts (11, 12). This underscores the need for more reliable, non-invasive tools to better stratify cancer risk, particularly in patients with low or intermediate PSA levels.

Emerging evidence supports the relevance of inflammation in cancer development and progression (13, 14). Inflammatory markers such as neutrophil-to-lymphocyte ratio (NLR), platelet-to-lymphocyte ratio (PLR), and monocyte-to-lymphocyte ratio (MLR) have been explored as diagnostic and prognostic indicators in various malignancies (15–18). Among these, MLR has demonstrated relatively stronger associations in certain clinical contexts (19).

Given these observations, the present study aims to evaluate the diagnostic utility of combining PSAD with MLR to improve PCa detection in patients presenting with PSA levels between 4.0 and 20.0 ng/mL.

## Method and materials

### Patient selection

A total of 80 patients with PSA levels ranging from 4.0 to 22 ng/mL were included in this retrospective study conducted between October 2016 and October 2022 at the Urology Department of the People’s Hospital of Xinjiang Uygur Autonomous Region, China. The size of the prostate was assessed using suprapubic ultrasonography (SU). All patients underwent ultrasound-guided prostate biopsy as PSA levels elevated. The prostate biopsy specimens were reviewed and diagnosed by pathologists at the pathology department of the hospital. All blood samples were collected on the second day after hospital admission. Pre-biopsy complete blood counts and PSA levels were obtained from the hospital laboratory. The prostate volume (PV) and parameters were calculated by the following formulae: PV = π/6 × width × length × height. NLR = absolute neutrophil count divided by the absolute lymphocyte count taken from full blood count results. MLR = ratio of the absolute peripheral blood monocyte and lymphocyte counts. PLR = absolute platelet and lymphocyte counts, respectively.

GraphPad Prism (GraphPad Software, Inc., San Diego, CA, USA) was used for the statistical analysis. Receiver operating characteristic (ROC) curve was used to analyze and evaluate the efficacy of NLR, MLR, PLR, f/tPSA, PSAD, and combined diagnosis for PCa. The Youden index was conducted to determine the optimal cutoff points for significant continuous variables. Then, the ROC-derived area under the curve (AUC), sensitivity, and specificity of the markers with their respective 95% confidence interval (CI) were computed to assess the diagnostic accuracy. Multiple logistic regression was used to determine if the combination of PSAD and inflammatory markers could improve the diagnostic efficacy.

Positive predictive value (PPV) and negative predictive value (NPV) were calculated based on sensitivity, specificity, and assumed prevalence rates of prostate cancer using the following formulae: PPV = (Se × prevalence)/[(Se × prevalence) + (1 - Sp) × (1-prevalence)]. NPV = (Sp × (1-prevalence))/[(1-Se) × prevalence + Sp × (1-prevalence)].

Due to the limited sample size, PPV and NPV were modeled across a range of assumed prevalence values to reflect possible variations in clinical settings.

Unpaired two-tailed Student’s *t*-test was used to define differences between two groups. A two-sided *p*-value of <0.05 was considered statistically significant.

## Results

### Baseline patient characteristic

A total of 80 patients with a median age of 66.7 years with 4.3–22 ng/mL of total PSA (tPSA) were enrolled in the study. PCa was diagnosed in 55 men and BPH was diagnosed in 25 men by prostate biopsy. The baseline clinical and pathological characteristics of all patients are depicted in **Table 1**.

**Table 1:** Patient characteristics.

Number, (n)	80
Mean age (range), yr	66.7 (42~88)
Median tPSA (range), ng/ml	10.93 (4.37~22.2)
Median fPSA (range), ng/ml	1.82 (0.33~6.06)
Median PV (range), ml	57.19 (9.5~202.6)
Pathologic Diagnosis, (n)	
Malignant	55
Benign	25

### Laboratory and suprapubic ultrasound findings

**Figure 1:**
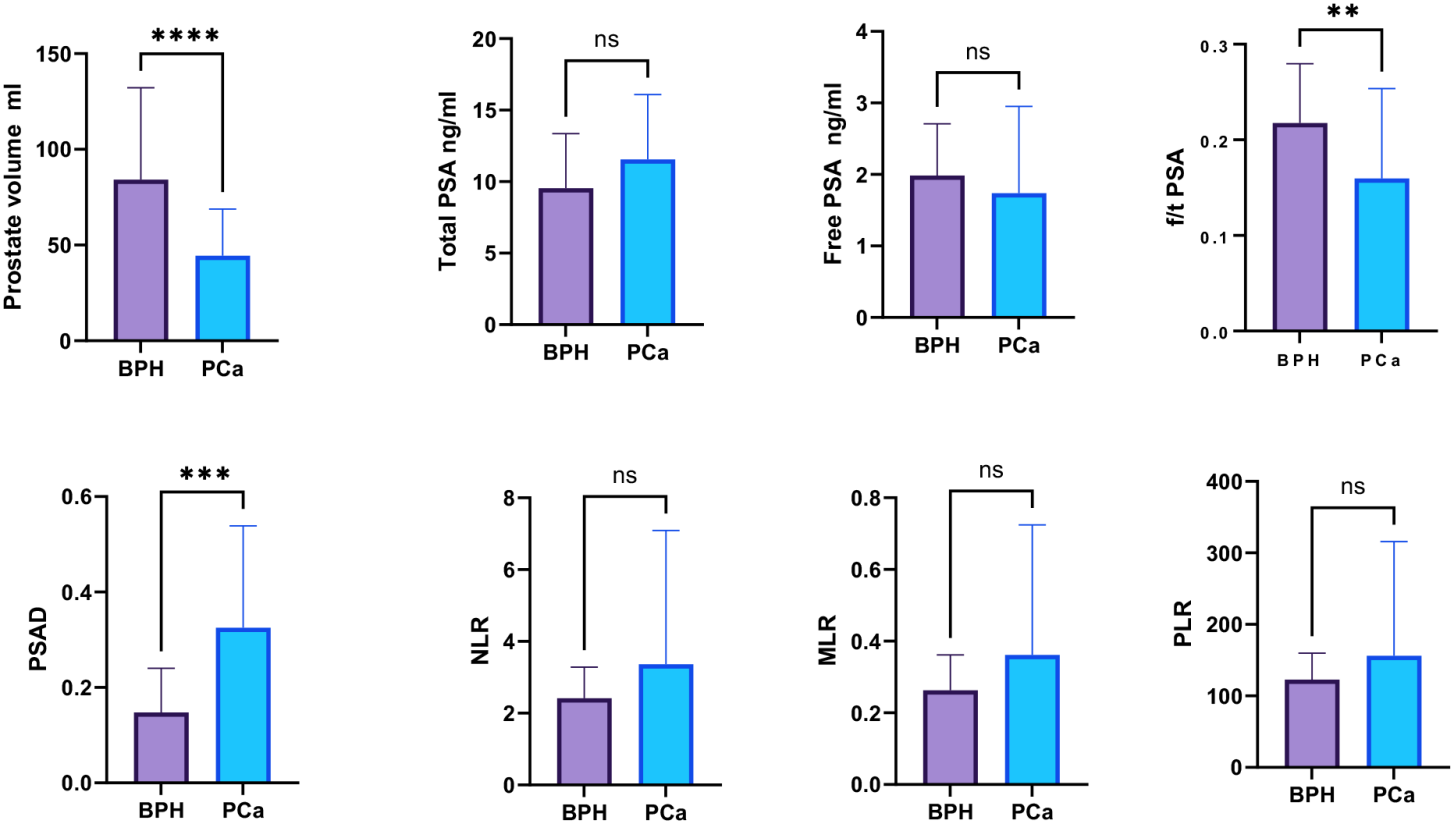
Comparison of the inflammatory markers NLR, PLR, and MLR between PCa and BPH patients. “ns”: not significant, “*”: p < 0.05, “**”: p < 0.01, “***”: p < 0.001, “****”: p < 0.0001.

The laboratory parameters and their comparison between PCa and BPH are depicted in **Table 2**. Patients with PCa were slightly older (median age of 69 vs. 61.56) with non-significantly differing tPSA and free PSA (fPSA) but substantially lower PV from patients with BPH (*p* < 0.0001). Conversely, the PSAD values were higher than in patients with BPH. The percent-free PSA (free-to-total f/tPSA) differed significantly (*P* < 0.001) between PCa patients and BPH patients as well. There was no statistical significance of NLR, MLR, and PLR between PCa patients and BPH patients (**Figure 1**).

**Table 2:** Comparisons of laboratory parameters and suprapubic ultrasonography (SU) measurements of the prostate sizes between PCa patients and BPH patients.

Characteristic	BPH n=25	PCa n=55	*P Value*
	Mean±SE	Mean±SE	
WBC, ×10^9^/L	7.23 ± 0.419	6.99 ± 0.288	0.6427
Neutrophils, ×10^9^/L	4.49± 0.299	5.83 ± 1.340	0.5051
Lymphocytes, ×10^9^/L	1.98 ± 0.137	1.96 ± 0.230	0.9496
Monocytes, ×10^9^/L	0.50 ± 0.040	0.583 ± 0.082	0.4967
Platelet, ×10^9^/L	227.4 ± 12.70	220.87 ± 8.289	0.6642
tPSA	9.54 ± 0.765	11.56 ± 0.611	0.0557
fPSA	1.98 ± 0.145	1.74 ± 0.164	0.3512
Median Prostate Volume, ml	84.20 ± 9.60	44.45 ± 3.349	<0.0001
Median PSAD	0.15 ± 0.018	0.33 ± 0.029	0.0002

### Diagnostic performance of laboratory parameters

**Figure 2:**
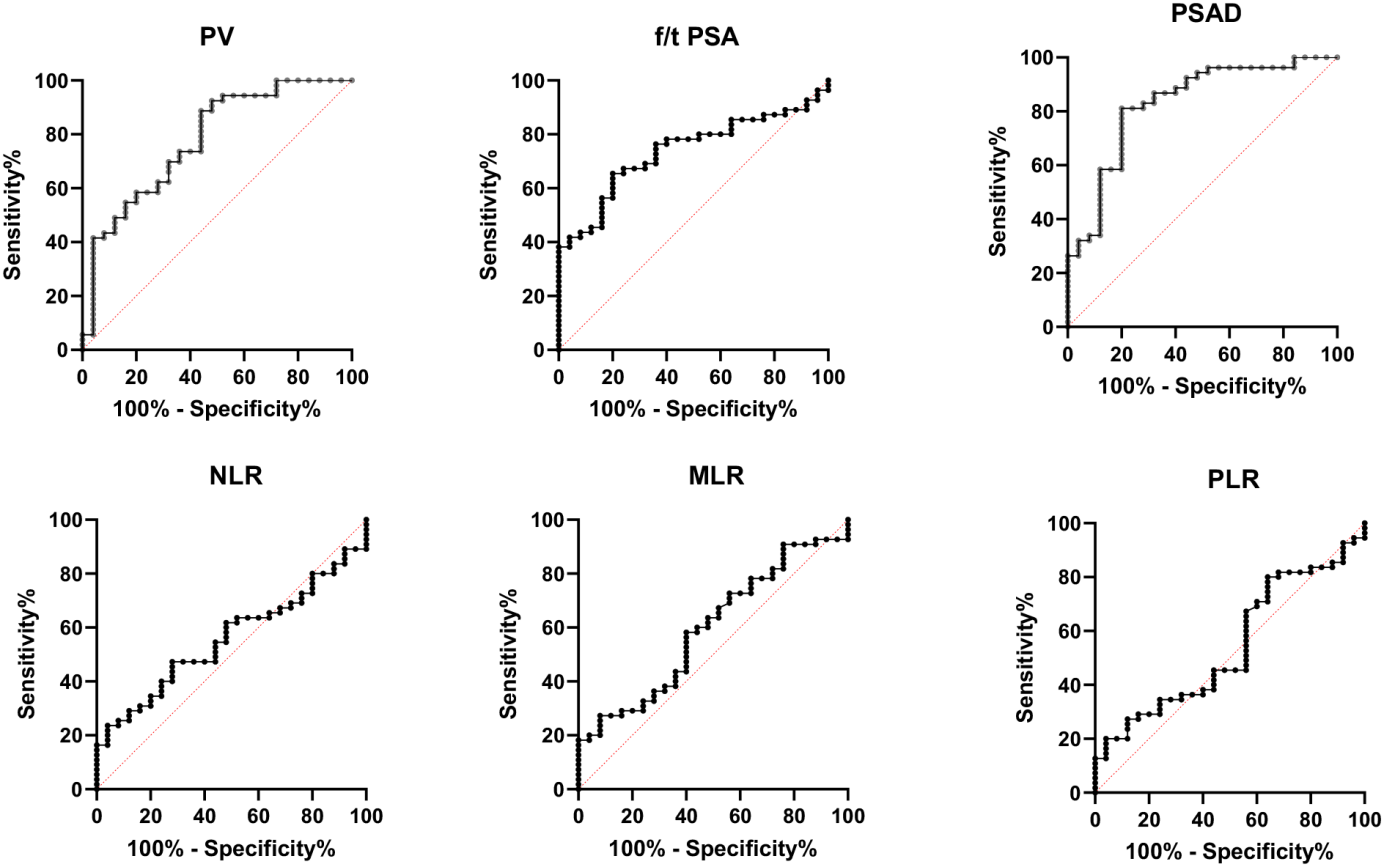
Receiver operating characteristic (ROC) curve analysis for the diagnosis of prostate cancer using percent-free PSA (f/t PSA), prostate volume (PV), prostate-specific antigen density (PSAD), neutrophil-to-lymphocyte ratio (NLR), monocyte-to-lymphocyte ratio (MLR), and platelet-to-lymphocyte ratio (PLR). PSAD demonstrated the highest diagnostic performance (AUC = 0.8294), followed by PV (AUC = 0.777) and f/t PSA (AUC = 0.728). In contrast, the inflammatory markers NLR (AUC = 0.557), MLR (AUC = 0.594), and PLR (AUC = 0.543) showed poor predictive value for prostate cancer discrimination.

The values of tPSA, f/tPSA, and PSAD in the diagnosis of BPH and PCa were analyzed by calculation of AUC under the ROC, and the Youden index was calculated to set the best cutoff value. The optimal cutoff was defined as the value with the highest Youden index. Among all individual analyses, the PSAD showed good sensitivity of 81.13% and specificity of 80% with a cutoff >0.188843 for diagnostic discrimination in PCa (AUC = 0.8294, 95% CI: 0.85–0.91, *p* < 0.0001), while the prostate volume (AUC = 0.777, 95% CI: 0.85–0.91, *p* < 0.0001) and f/t PSA (AUC = 0.728, 95% CI: 0.85–0.91, *p* < 0.001) showed fair predictive ability for PCa as well (**Table 3**, **Figure 2**).

**Table 3:** Diagnostic value of PV, f/tPSA, PSAD, and inflammatory biomarkers for PCa.

Variables	AUC	Cutoff	Sensitivity	Specificity	95% confidence interval	*P*-value
PV	0.7774	49.2003	69.81%	68%	0.664622–0.890095	<0.0001
f/t PSA	0.7375	0.1798	69.09%	68%	0.629619–0.845290	0.0007
PSAD	0.8294	0.1888	81.13%	80%	0.728144–0.930724	<0.0001
MLR	0.5945	0.3917	27.27%	92%	0.463456–0.725635	0.1772
NLR	0.5571	3.9452	23.64%	96%	0.431349–0.6828833	0.4152
PLR	0.5437	96.5915	80%	36%	0.410160–0.677113	0.5334
PSAD + MLR	0.8717		87.27% (PP)	78.26% (NP)	0.7667–0.9586	<0.0001
PSAD + NLR	0.8574		83.93% (PP)	72.73% (NP)	0.7616–0.9531	<0.0001
PSAD + PLR	0.8460		82.14% (PP)	68.18% (NP)	0.7491–0.9430	<0.0001

PP, positive predictive value; NP, negative predictive value.

**Figure 3:**
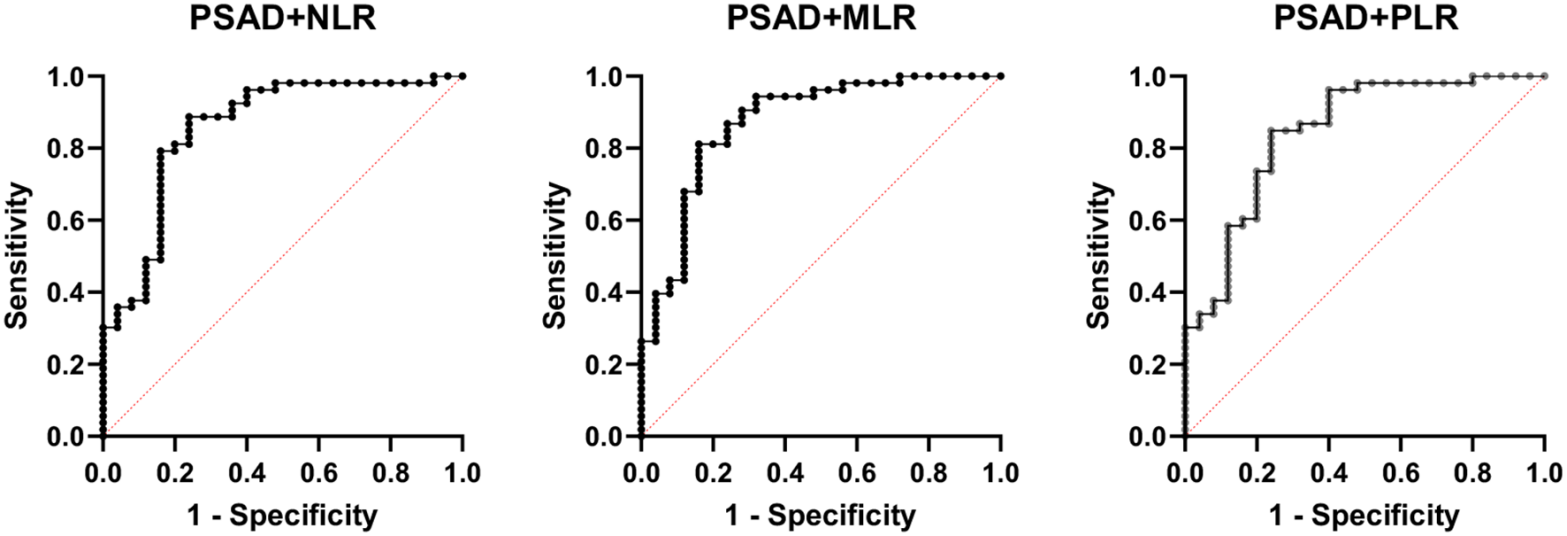
Receiver operating characteristic (ROC) curve analysis showing the diagnostic performance of prostate-specific antigen density (PSAD) combined with inflammatory markers—neutrophil-to-lymphocyte ratio (NLR), monocyte-to-lymphocyte ratio (MLR), and platelet-to-lymphocyte ratio (PLR)—for prostate cancer detection. Among the combinations, PSAD + MLR demonstrated the highest diagnostic accuracy with an AUC of 0.8717, followed by PSAD + NLR (AUC = 0.8417) and PSAD + PLR (AUC = 0.8421). These results suggest that combining PSAD with MLR notably improves the discriminatory ability for prostate cancer compared to PSAD alone.

**Figure 4:**
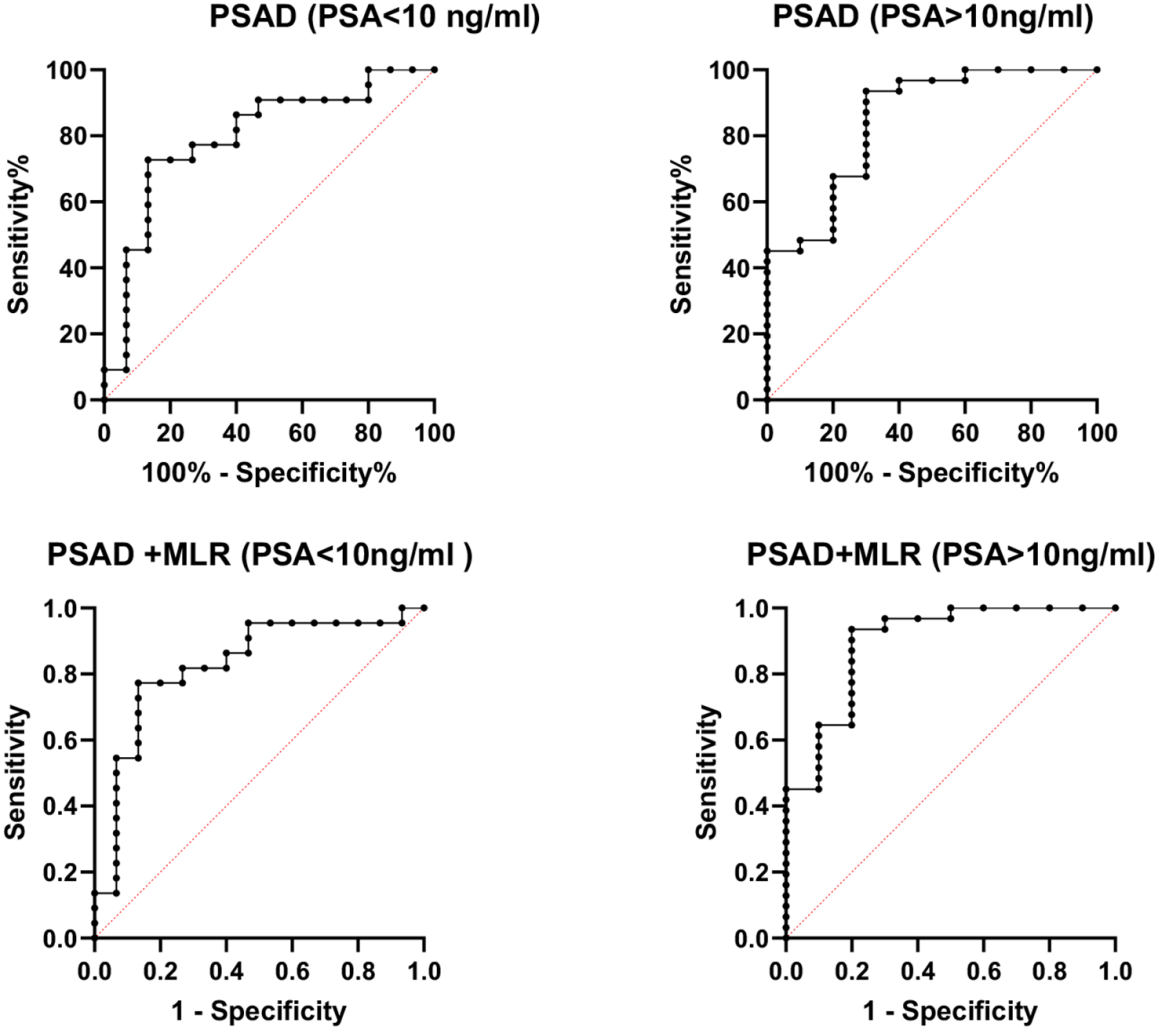
Receiver operating characteristic (ROC) curve analysis of combined prostate-specific antigen density (PSAD) and monocyte-to-lymphocyte ratio (MLR) stratified by PSA levels for prostate cancer diagnosis. The diagnostic performance was assessed in two PSA subgroups: 4–10 ng/mL (AUC = 0.8484) and 10–22 ng/mL (AUC = 0.8968). The combination of PSAD and MLR showed improved accuracy in detecting prostate cancer across both PSA ranges.

However, the inflammatory markers NLR, MLR, and PLR showed poor predictive ability for PCa (AUC = 0.557, 0.594, and 0.543) (**Table 3**, **Figure 2**). Combing the PSAD with inflammatory markers, particularly combing the PSAD with MLR, likewise substantially strengthened the AUC value (0.8717) for the detection of prostate cancer with good positive (87.27%) and negative predictive values (78.26%). The sensitivity and specificity of the PSAD + MLR combination were 87.27% and 78.26%, respectively, further supporting its diagnostic accuracy (**Table 3**). The positive predictive values were superior to the negative predictive values (**Table 3**, **Figure 3**). Subsequently, we categorized the patients to either the cohort of PSA 4–10 ng/mL or cohort of PSA 10–22 ng/mL (**Table 4**). The AUCs for PSAD and PSAD + MLR for predicting PCa in patients with a PSA level of 4.0–10 or 10–22 ng/mL were 0.7969, 0.8484, 0.8273, and 0.8968, respectively (**Table 5**, **Figure 4**).

**Table 4:** Pathological characteristics of patients according to serum PSA levels.

Characteristic	PSA 4-10ng/ml (n=37)	PSA 10-22 ng/ml (n=43)
BPH	n= 15	n= 10
PCa	n=22	n=33
Gleason Score 6	n=6	n=7
Gleason Score 7	n=10	n=14
Gleason Score ≥ 8	n=6	n=12

**Table 5:** Diagnostic performance for prostate cancer based on different PSA levels.

PSA range	Model	AUC (95% CI)	Sensitivity	Specificity	PPV	NPV	*P*-value
4–10 ng/mL	PSAD	0.797 (0.644–0.950)	72.73%	86.67%	88.9%	68.4%	0.0024
10–22 ng/mL	PSAD	0.848 (0.703–0.993)	93.55%	70%	91.1%	76.7%	0.001
4–10 ng/mL	PSAD + MLR	0.827 (0.684–0.971)	—	—	81.81%	73.33%	0.0008
10–22 ng/mL	PSAD + MLR	0.897 (0.775–1.000)	—	—	90.91%	87.50%	0.0002

PPV, positive predictive value; NPV, negative predictive value; Sen, sensitivity; Spe, specificity.

## Discussion

Prostate cancer (PCa) is the second most common cancer among men and remains a significant cause of morbidity and mortality worldwide (20). Although prostate-specific antigen (PSA) has been widely used for early detection, its low specificity—especially in the “gray zone” (4–10 ng/mL)—may lead to missed diagnoses or overdiagnosis. PSA levels can be elevated due to benign prostatic hyperplasia (BPH), prostatitis, or other non-malignant conditions, making sole reliance on PSA insufficient.

Several PSA-derived metrics, including total PSA (tPSA), free PSA (fPSA), free-to-total PSA ratio (f/t PSA), and PSA density (PSAD), have been proposed to improve the diagnostic precision (21). In our study, PSAD demonstrated superior sensitivity and specificity to identify PCa among patients with PSA levels between 4 and 20 ng/mL, showing greater discriminatory ability than tPSA, fPSA, and f/t PSA. This aligns with previous findings by Verma et al., who reported higher AUC values for PSAD in patients with PSA >10 ng/mL compared to those in the gray zone (0.72 vs. 0.61) (22). In our cohort, we found AUC values of 0.848 for PSA >10 ng/mL and 0.797 for PSA <10 ng/mL, further confirming the usefulness of PSAD across different PSA ranges.

In addition to PSA-related markers, systemic inflammation has emerged as an important contributor to tumor development, progression, and metastasis (23). Inflammatory indices such as the neutrophil-to-lymphocyte ratio (NLR), monocyte-to-lymphocyte ratio (MLR), and platelet-to-lymphocyte ratio (PLR) have been explored as potential indicators to distinguish between benign and malignant conditions (24). Although in our study these inflammatory markers had poor diagnostic value individually, MLR showed a relatively higher predictive value compared to PLR and NLR. Importantly, the combination of PSAD and MLR significantly enhanced the diagnostic accuracy compared to PSAD alone. The combined model achieved AUCs of 0.848 and 0.897 in the PSA 4–10 and 10–22 ng/mL subgroups, respectively, indicating that PSAD + MLR may be useful even in patients with borderline PSA levels. This suggests the potential of PSAD + MLR as a useful adjunct to guide biopsy decisions, particularly to support active surveillance strategies and reduce unnecessary procedures, especially in borderline PSA cases. This aligns with growing efforts to tailor PCa diagnosis and management based on risk stratification.

While our study did not include prostate magnetic resonance imaging (MRI), we acknowledge that multiparametric MRI (mpMRI), particularly the Prostate Imaging Reporting and Data System (PiRADS), has become an important tool in PCa detection and biopsy decision-making. Additionally, the International Society of Urological Pathology (ISUP) grade group system is widely used to evaluate biopsy pathology. Future studies should integrate these radiologic and pathologic assessments with PSAD and MLR to explore more comprehensive, multi-modal diagnostic models.

We recognize that this study has several limitations. It is a retrospective, single-center analysis with a limited sample size (*n* = 80), which may restrict the generalizability of the results. Additionally, prostate volume was measured using suprapubic ultrasonography (SU), a widely accessible but less accurate method compared to transrectal ultrasound (TRUS) or multiparametric MRI (mpMRI). Volume estimates may have been subject to operator-dependent variability, which could affect PSAD calculations. Future studies should incorporate TRUS or MRI-based volume measurements to improve the precision of PSAD assessments. Our study also lacked internal and external validation. Without validation using independent datasets or multicenter cohorts, the robustness of the PSAD + MLR model remains uncertain. Future work should employ internal methods (e.g., cross-validation or bootstrapping) and pursue external validation to assess generalizability. Integrating machine learning algorithms may also help optimize the predictive model while minimizing overfitting. Another important limitation is the absence of advanced diagnostic tools such as mpMRI, which offers high-resolution imaging for lesion localization, and PiRADS scoring, which guides biopsy decisions. Furthermore, we did not incorporate ISUP grade group pathology data, which is essential for comprehensive tumor grading. Future studies should integrate these modalities to further evaluate the combined value of PSAD and MLR within a multi-modal diagnostic framework. Finally, although our model showed a high positive predictive value (PPV), the small sample size limited the accurate calculation of subgroup-specific sensitivity and specificity. Larger prospective cohorts will be necessary to fully characterize these parameters and assess clinical applicability across diverse patient populations.

In conclusion, our study suggests that PSA density (PSAD) has better diagnostic performance than tPSA or f/t PSA for detecting prostate cancer, especially in men with PSA levels in the 4–10 ng/mL “gray zone.” Moreover, combining PSAD with MLR improves the diagnostic accuracy and may serve as a useful, low-cost adjunct to identify patients at a higher risk of PCa. This combined marker model could contribute to reducing unnecessary prostate biopsies, supporting active surveillance, and minimizing overtreatment in clinical practice. Future studies incorporating transrectal ultrasound, mpMRI (PiRADS), and ISUP grading will be essential to further validate and optimize this diagnostic approach.

## Data Availability

The original contributions presented in the study are included in the article/supplementary material. Further inquiries can be directed to the corresponding author.
